# Single M-Line Is as Reliable as Multiple M-Line Ultrasound for Carotid Artery Screening

**DOI:** 10.3389/fphys.2021.787083

**Published:** 2021-12-20

**Authors:** Afrah E. F. Malik, Tammo Delhaas, Bart Spronck, Ronald M. A. Henry, Jayaraj Joseph, Coen D. A. Stehouwer, Werner H. Mess, Koen D. Reesink

**Affiliations:** ^1^Department of Biomedical Engineering, Maastricht University Medical Center, Maastricht, Netherlands; ^2^CARIM School for Cardiovascular Diseases, Maastricht University Medical Center, Maastricht, Netherlands; ^3^Department of Internal Medicine, Maastricht University Medical Center, Maastricht, Netherlands; ^4^Healthcare Technology Innovation Centre, Indian Institute of Technology Madras, Chennai, India; ^5^Department of Clinical Neurophysiology, Maastricht University Medical Centre, Maastricht, Netherlands

**Keywords:** arterial stiffness, common carotid artery, echo tracking, reproducibility, vascular risk management, vascular ultrasound

## Abstract

**Purpose:** Carotid artery properties can be evaluated with high accuracy and reproducibility using multiple M-line ultrasound. However, the cost of multiple M-line-based imaging modalities and the extensive operator expertise requirements hamper the large-scale application for arterial properties assessment, particularly in resource-constrained settings. This study is aimed to assess the performance of a single M-line approach as an affordable and easy-to-use alternative to multiple M-line imaging for screening purposes.

**Methods:** We used triplicate longitudinal common carotid artery (CCA) ultrasound recordings (17 M-lines covering about 16 mm, at 500 frames per second) of 500 subjects from The Maastricht Study to assess the validity and reproducibility of a single against multiple M-line approach. The multiple M-line measures were obtained by averaging over all available 17 lines, whereas the middle M-line was used as a proxy for the single M-line approach.

**Results:** Diameter, intima-media thickness (IMT), and Young's elastic modulus (YEM) were not significantly different between the single and multiple M-line approaches (*p* > 0.07). Distension and distensibility coefficient (DC) did differ significantly (*p* < 0.001), however, differences were technically irrelevant. Similarly, Bland-Altman analysis revealed good agreement between the two approaches. The single M-line approach, compared to multiple M-line, exhibited an acceptable reproducibility coefficient of variation (CV) for diameter (2.5 vs. 2.2%), IMT (11.9 vs. 7.9%), distension (10 vs. 9.4%), DC (10.9 vs. 10.2%), and YEM (26.5 vs. 20.5%). Furthermore, in our study population, both methods showed a similar capability to detect age-related differences in arterial stiffness.

**Conclusion:** Single M-line ultrasound appears to be a promising tool to estimate anatomical and functional CCA properties with very acceptable validity and reproducibility. Based on our results, we might infer that image-free, single M-line tools could be suited for screening and for performing population studies in low-resource settings worldwide. Whether the comparison between single and multiple M-line devices will yield similar findings requires further study.

## Introduction

Cardiovascular (CV) diseases are the leading cause of global mortality. An estimated global CV mortality of 17.9 million was recorded in 2019 (World Health Organization, 2021), with lower- and middle income (LMI) countries accounting for three times more CV cases than high-income countries (Jagannathan et al., 2019). Compared to previous decades, there has been a downward trend in CV mortality in developed countries. However, the rate of CV deaths in LMI countries has remained constant, mainly driven by the low detection rate of early-stage CV diseases in these regions (Jagannathan et al., 2019). Early detection of CV diseases facilitates early intervention and is, hence, necessary to reduce the global burden of CV diseases.

Arterial stiffness is recognized as an important predictor of CV diseases at their early stages. By contributing to elevated systolic blood pressure and increased cardiac afterload, an increased arterial stiffness causes CV diseases (Engelen et al., 2015). Increased arterial stiffness is associated with an enhanced risk of coronary heart disease, cerebrovascular events, and all-cause mortality (Laurent et al., 2001; Mattace-Raso et al., 2006; Karras et al., 2012; Steinbuch et al., 2016).

Carotid-femoral pulse wave velocity is considered to be the gold-standard measurement of arterial stiffness (Laurent et al., 2006). It represents the average stiffness over a long trajectory of the arterial tree. This trajectory, however, involves both central elastic and more muscular arteries (Engelen et al., 2015). The stiffness of these arteries may differently relate to CV diseases (Engelen et al., 2015). In addition, atherosclerotic plaques may result in a local modification of the stiffness, while the rest of the arterial tree remains intact (Hermeling, 2009). Therefore, it might be of particular interest to separately measure the stiffness of different arteries. Common carotid artery (CCA) stiffness, defined as distensibility coefficient (DC), is an independent predictor of CV events and all-cause mortality (Alan et al., 2003; Godia et al., 2007; Yuan et al., 2016; Joseph et al., 2020b).

The common practice of estimating local arterial stiffness involves measuring the instantaneous change in carotid diameter—termed distension—by means of ultrasound echo tracking. Combined with a local pulse pressure (PP) estimate, one obtains DC and Young's elastic modulus (YEM). The evaluation of local carotid stiffness requires accurate and precise tracking of the arterial wall. Conventionally, the tracking is based either on the edges of the arterial wall from a B-mode video recording or on the phase of multiple M-line recording. While scanners based on the former technique are limited by the temporal resolution of B-mode recordings, scanners based on phase tracking are costly and depend on the expertise of the operator. This limits their applicability to a small number of specialized hospitals. Both types of scanners, therefore, do not meet the pressing need for a screening tool in low-resource areas. Devices based on a single M-line (Joseph et al., 2020a) are affordable and accessible tools that are promising for diagnosis and screening in LMI countries.

The question that can be asked is whether carotid properties obtained with single M-line-based scanners are as accurate and reproducible as those obtained with multiple M-line-based scanners. Therefore, the aim of this study was to compare the performance of a single against a multiple M-line approach. For this purpose, we exploited existing multiple M-line image-based recordings and used the middle M-line as a proxy for single M-line-based devices. More specifically, we: (1) assessed the validity and compared the reproducibility of our proxy against a multiple M-line approach, and (2) compared the capacity of both methods to determine age-associated changes in stiffness.

## Materials and Methods

### Study Subjects

In this study, we utilized data from The Maastricht Study, an ongoing, observational, prospective, population-based cohort study. It includes residents of the southern part of the Netherlands aged between 40 and 75 years. Its rationale, methodology, and design have been described in Schram et al. (2014). Briefly, the study focuses on the causation, pathophysiology, complications, and comorbidities of type 2 diabetes. The present study includes 500 participants randomly selected from over 7,000 subjects recruited in the first round of the study. In all selected subjects, three repeated measurements were performed (in accordance with the study protocol). Subject characteristics are presented in Table 1. The Maastricht Study has been approved by the institutional medical ethical committee and the Netherlands Health Council. Written informed consent was obtained from all participants prior to the study.

**Table 1:** Characteristics of the study population.

*N*	499
Age (years)	60 ± 8
Men (%)	52
Women (%)	48
BMI (kg/*m*^2^)	26 ± 4
Weight (kg)	78 ± 15
Height (cm)	172 ± 9
Systolic blood pressure (mmHg)	127 ± 14
Diastolic blood pressure (mmHg)	75 ± 7
Pulse pressure (mmHg)	52 ± 10
Heart rate (beats/min)	62 ± 9
History of cardiovascular disease (%)	15.4

*Values are presented as mean ± SD or percentage as appropriate*.

### Data Acquisition

All vascular measurements were performed by trained technicians in temperature-controlled rooms (21–23°C) as described in Wijnands et al. (2015) and Zhou et al. (2018). Participants were asked to abstain from smoking and caffeine-containing beverages 3 h prior to the study (Wijnands et al., 2015). Vascular measurements were performed in the supine position after 10 min of rest. Repeated longitudinal ultrasound measurements of the left CCA were performed at least 1 cm proximally from the bifurcation using a Mylab70 scanner (Esaote Europe, Maastricht, The Netherlands) with a 7.5 MHz linear array transducer. During ultrasound measurements, systolic and diastolic pressure of the brachial artery was measured with an oscillometric blood pressure monitor (Accutorr Plus, Datascope Inc., Montvale, NJ, USA). Next, PP was calculated as the difference between systolic and diastolic blood pressures. The average of pressure measurements performed at 5-min intervals during the vascular examination (lasting ~45 min) was considered for further analysis (Geijselaers et al., 2016).

### Arterial Wall Tracking

The scanner enabled the recording of radiofrequency (RF) signals in fast B-mode with a frame rate of 498 Hz. Although this very high frame rate enables high precision in wall tracking (Meinders et al., 2003), it comes at the cost of a reduced number of recording positions in the longitudinal plane (17 M-lines, separated by 0.96 mm, covering 16.32 mm). This results in a reduction of the longitudinal resolution while retaining information along the entire length of the imaged vessel. For off-line processing, RF signals were fed into a PC-based acquisition system that was coupled with the scanner (ART.LAB, Esaote Europe B.V. Maastricht, the Netherlands) (Wijnands et al., 2015; Geijselaers et al., 2016). RF signals were sampled at 50 MHz. During measurements, a video was depicted on the screen of the PC and an online tracking algorithm showed real-time instantaneous displacement of the arterial wall to assist the operator in orienting the ultrasound probe and obtaining high-quality RF signals. The RF signals were processed in MATLAB (version 7.5, Mathworks, Natick, MA, USA) using a wall-tracking system (WTS), which follows steps that were previously described in Hoeks et al. (1990, 1997). In brief, intima-media thickness (IMT) is assessed in the far wall by estimating the difference in position between the leading edge of the lumen intima echo and the leading edge of the media-adventitia echo during diastole (Willekes et al., 1999). Diameter is defined as the distance between the trailing edge of the anterior and leading edge of the posterior wall media-adventitia echoes obtained during diastole. The distension waveforms were obtained by tracking the displacement of the media-adventitia transition in the anterior and posterior walls. This was achieved by employing an efficient and commonly used complex cross-correlation model on the phase of the corresponding RF signal (Brands et al., 1997; Meinders et al., 2001; Steinbuch et al., 2016).

**Figure 1:**
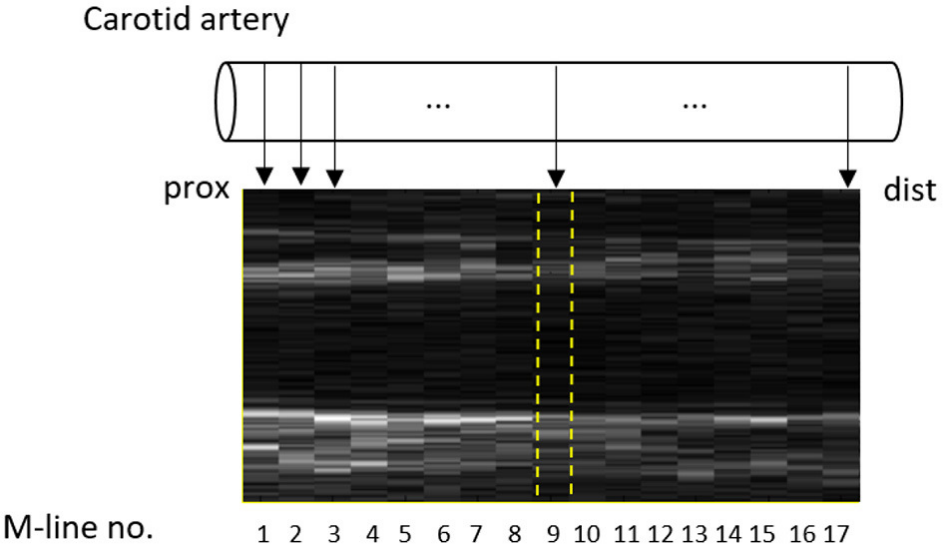
Fast B-mode ultrasound recording of the common carotid artery with the 17 M-lines indicated. The middle M-line (no. 9) was selected as a proxy for a single M-line device and is indicated by dashed (yellow) lines.

The WTS performs the abovementioned procedure and estimates the wall properties for each M-line separately. Conventionally, the average over all individual M-lines is considered for each parameter (Meinders and Hoeks, 2004). We refer to this method as the multiple M-line approach. As described in the introduction, we used the middle M-line as a proxy for an image-free single M-line device, which is referred to as the single M-line approach (Figure 1).

The simultaneous assessment of the geometrical parameters, such as diameter, wall thickness, and changes in diameter, in combination with PP, enables the quantification of the local mechanical characteristics of the vessel wall. DC is the relative change in vessel cross-sectional area for a given increase in pressure (Hoeks et al., 1990). YEM reflects the stiffness of a vessel as a function of diastolic diameter, a relative increase in diameter, IMT, and PP (Hoeks et al., 1997). These indices are defined as (Zhou et al., 2018):


(1) $$\begin{array}{lll} \text{DC} & = & \;\frac{2D\Delta D+\Delta D^{2}\;}{{\text{PP}}^{*}D^{2}} \end{array}$$

(2) $$\begin{array}{rcl} \text{YEM} & = & D/\left({\text{IMT}}^{*}\text{DC}\right) \end{array}$$

where *D* is the diastolic diameter; Δ*D* is the distension; PP is the brachial pulse pressure; and IMT is the intima-media thickness.

### Statistical Analyses

Statistical analyses were performed using SPSS version 27 (SPSS, Chicago, IL, USA). To validate the single M-line against the multiple M-line approach, a comparative analysis was performed. The multiple M-line approach was considered as a reference against which we evaluated the performance of the single M-line approach. This analysis included geometrical parameters and mechanical characteristics of the CCA. The validity of the single M-line approach was evaluated in two ways. First, Bland-Altman analysis was employed to assess the agreement between the two methods. Subsequently, a paired *t*-test was employed to assess the differences between single and multiple M-line approaches. A two-sided *p*-value < 0.05 was considered statistically significant.

The reproducibility of the single M-line approach was assessed using the intra-subject SD of repeated measurements. In addition, the coefficient of variation (CV) was calculated as [(intra-subject SD/group mean) ×100%]. The computation of within-subject and between-subject SDs has been described previously in Rodbard (1974). Composite SD (SD_comp_), which represents the expected between-subject variation for a sample of repeated measurements (*n*_rep_), is evaluated using the within-subject SD (SD_w_) and between-subject SD (SD_b_) using the formula:


(3) $$\begin{array}{l} {\text{SD}}_{\text{comp}}=\;\sqrt{{{\text{SD}}_{\text{b}}}^{2}+\;\frac{{{\text{SD}}_{\text{w}}}^{2}}{n_{\text{rep}}}\;} \end{array}$$

Equation (3) estimates the expected composite SD for any number of repetitions, e.g., to be utilized for power calculations in future studies.

We considered the middle M-line of a fast B-mode recording to be representative of a single M-line used in an image-free screening tool. However, with an imageless device, the position and orientation of the single M-line relative to the CCA are less controlled. Therefore, we performed a sensitivity analysis exploring the performance of other single M-lines. Additionally, we explored the influence of averaging different quantiles of M-lines on the validity and reproducibility of carotid properties. This was achieved by employing an averaging window around M-line 9 with increasing width from 1 to 15 M-lines using 2-line steps.

Multiple studies have firmly established an age-associated increase in arterial stiffness (Avolio et al., 1983, 1985; Kawasaki et al., 1987). Hence, we extended our evaluation by assessing the capability of the single M-line approach to detect the stiffness increase with age within our study population. To this end, study subjects were divided into two age groups (<60 and ≥60 years), and an independent sample *t*-test was employed to test for differences in stiffness measures between age groups. A similar procedure was applied to the multiple M-line approach to allow comparison between the two methods. The capability of the two methods to detect the increase in stiffness was compared using repeated-measures ANOVA.

## Results

Among the 500 subjects included in the study, one patient was excluded from all statistical analyses due to motion artifacts in the corresponding recording.

### Validity and Reproducibility of the Single M-Line Approach

**Figure 2:**
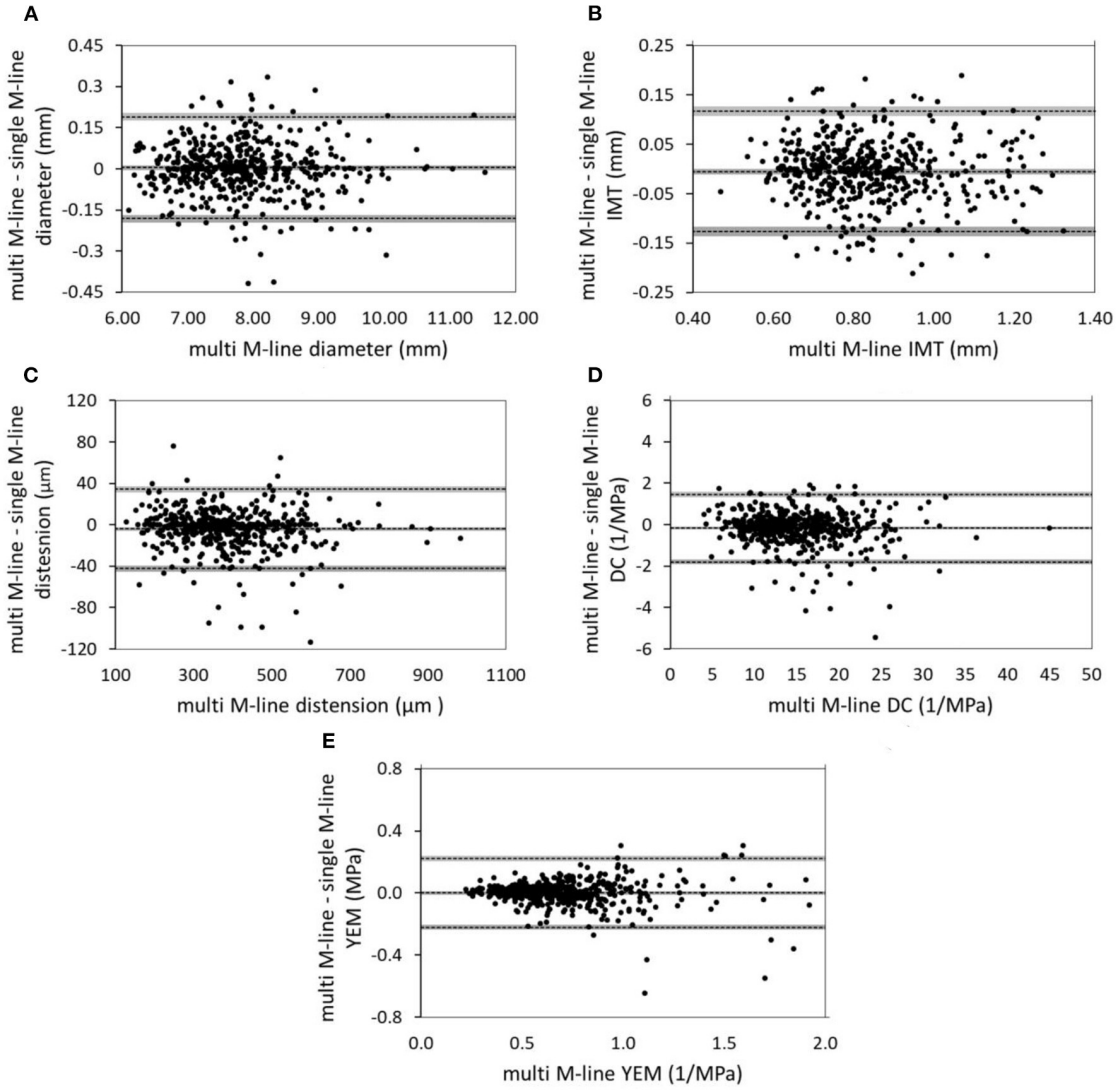
Bland-Altman plots show good agreement between single and multiple-M-line approaches in estimating **(A)** diameter, **(B)** IMT, **(C)** distension, **(D)** DC, and **(E)** YEM. The gray areas indicate the 95% CIs of the bias and the limits of agreement. IMT, intima media thickness; DC, distensibility coefficient; YEM, young's elastic modulus.

Reproducibility statistics were calculated based on three repeated measurements. These statistics that include the 25–75% CI obtained using bootstrapping are presented in Table 2. The composite SD was also computed using the within-subject and between-subject SDs. Although there was a significant difference between the intra-subject SDs obtained by the single and multiple M-line approaches, with the SD for multiple M-line being slightly lower (better), both tracking methods exhibited very low within-subject SD for carotid diameter (0.19 vs. 0.17 mm). The carotid diameter was similar for both single and multiple M-line approaches (7.79 ± 0.91 vs. 7.80 ± 0.90 mm, *p* = 0.34). Moreover, the Bland-Altman analysis shown in Figure 2A also revealed a good agreement between the single and multiple M-line approaches for estimating diameter (95% limits of agreement: −0.18 to 0.19 mm).

**Table 2:** Common carotid artery properties as determined by single and multiple M-line approaches.

			**Single M-line**	**Multiple M-line**	***p*-value**
Carotid diameter	Diameter (mm)		7.79 ± 0.91	7.80 ± 0.90	0.34
	Intra-subject	SD (mm)	0.19 (0.18–0.20)	0.17 (0.16–0.18)	<0.001
		CV (%)	2.5 (2.4–2.6)	2.2 (2.1–2.3)	
	Between subject	SD (mm)	0.90 (0.87–0.92)	0.89 (0.86–0.91)	
		CV (%)	11.5 (11.2–11.8)	11.4 (11.1–11.7)	
	Composite (*n*_rep_ = 3)	SD (mm)	0.90 (0.88–0.93)	0.89 (0.87–0.92)	
		CV (%)	11.6 (11.3–11.9)	11.5 (11.2–11.8)	
Carotid IMT	IMT (mm)		0.86 ± 0.20	0.85 ± 0.18	0.07
	Intra-subject	SD (mm)	0.10 (0.10–11)	0.07 (0.06–0.07)	<0.001
		CV (%)	11.9 (11.6–12.3)	7.9 (7.6–8.2)	
	Between subject	SD (mm)	0.18(0.17–0.19)	0.17 (0.17–0.18)	
		CV (%)	21.2 (20.1–22.0)	20.4 (19.4–21.2)	
	Composite (*n*_rep_ = 3)	SD (μm)	0.19 (0.18–0.20)	0.18 (0.17–0.19)	
		CV (%)	22.3 (21.8–23.5)	20.9 (20.2–21.9)	
Carotid distension	Distension (μm)		389 ± 132	385 ± 128	<0.001
	Intra-subject	SD (μm)	39 (37–40)	36 (35–37)	<0.001
		CV (%)	10.0 (9.6–10.3)	9.4 (9.0–9.7)	
	Between subject	SD (μm)	129 (125–132)	126 (122–129)	
		CV (%)	33.2 (32.3–34.0)	32.7 (31.8–33.5)	
	Composite (*n*_rep_ = 3)	SD (μm)	131 (127–134)	127 (123–131)	
		CV (%)	33.7 (33.0–34.7)	33.1 (32.4–34.1)	
Carotid distensibility coefficient	DC (1/MPa)		15.3 ± 5.5	15.2 ± 5.3	<0.001
	Intra-subject	SD (1/MPa)	1.67 (1.61–1.72)	1.55 (1.49–1.60)	<0.001
		CV (%)	10.9 (10.5–11.2)	10.2 (9.9–10.6)	
	Between subject	SD (1/MPa)	5.32 (5.15–5.47)	5.17 (5.00–5.31)	
		CV (%)	34.8 (33.8–35.6)	34.2 (33.2–35.0)	
	Composite (*n*_rep_ = 3)	SD (1/MPa)	5.41 (5.24–5.55)	5.24 (5.07–5.38)	
		CV (%)	35.4 (34.7–36.5)	34.7 (34.0–35.8)	
Carotid YEM	YEM (MPa)		0.70 ± 0.33	0.71 ± 0.30	0.90
	Intra-subject	SD (MPa)	0.19 (0.18–0.20)	0.15 (0.13–0.16)	<0.001
		CV (%)	26.5 (24.7–27.9)	20.5 (18.7–21.8)	
	Between subject	SD (MPa)	0.32 (0.30–0.33)	0.29 (0.28–0.30)	
		CV (%)	44.4 (41.8–46.3)	41.0 (39.5–42.2)	
	Composite (*n*_rep_ = 3)	SD (MPa)	0.34 (0.32–0.35)	0.30 (0.29–0.32)	
		CV (%)	46.9 (45.6–50.1)	42.6 (41.9–44.7)	

*Values are expressed as mean ± SD. p-value based on paired sample t-test. Reproducibility statistics are presented that includes the 25–75% CI (obtained using bootstrapping) between parentheses. SD, standard deviation; CV, coefficient of variation; IMT, intima-media thickness; YEM, young's elastic modulus; DC, distensibility coefficient*.

The single M-line approach yielded a significantly higher intra-subject SD for IMT compared to multiple M-line (0.10 vs. 0.07 mm, *p* < 0.001). However, the Bland-Altman plot shown in Figure 2B demonstrates a good agreement between single and multiple M-line approaches for IMT (95% limits of agreement: −0.13 to 0.12 mm), with no significant bias between single and multiple M-line-derived IMT (0.86 ± 0.20 vs. 0.85 ± 0.18 mm, *p* = 0.07).

Compared to when using multiple M-lines, the single M-line approach resulted in a significantly higher intra-subject SD for distension (39 vs. 36 μm, *p* < 0.001) and DC (1.67 vs. 1.55 1/MPa, *p* < 0.001). Similarly, the single M-line method yielded clinically irrelevant but significantly higher distension and DC (389 ± 132 vs. 385 ± 128 μm, and 15.3 ± 5.5 vs. 15.2 ± 5.3 1/MPa, respectively, *p* < 0.001). Nevertheless, the Bland-Altman analysis (Figures 2C,D) revealed good agreement between the single and the multiple M-line approach (95% limits of agreement: −42 to 34 μm and −1.8 to 1.5 1/MPa for distension and DC, respectively).

The intra-subject SD of YEM obtained by the single M-line was significantly higher than that achieved with multiple M-lines (0.19 vs. 0.15 MPa, *p* < 0.001). However, the Bland-Altman plot shown in Figure 2E reveals good agreement between single and multiple M-line approaches for YEM (95% limits of agreement: −0.22 to 0.22 MPa). This finding is corroborated by their non-significant difference (0.70 ± 0.33 vs. 0.71 ± 0.30 MPa, *p* = 0.90).

### Sensitivity Analysis

**Figure 3:**
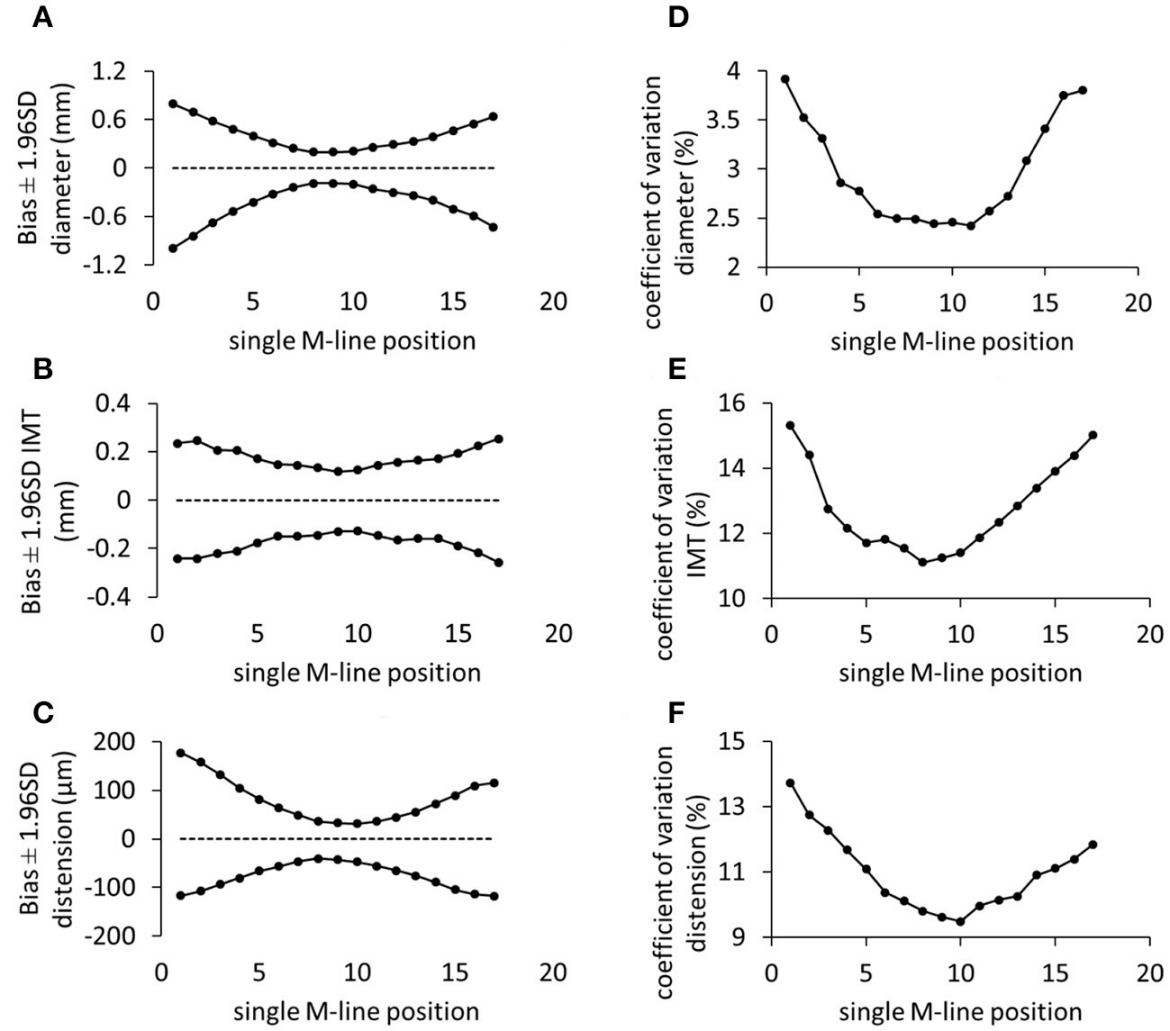
The performance of individual M-lines in terms of the agreement from the Bland-Altman analysis **(A–C)** and reproducibility coefficient of variation **(D–F)** for diameter, intima-media thickness (IMT), and distension, respectively. SD, standard deviation.

Figures 3A–C shows how the choice of the single M-line (among the 17 available) affects the 95% limits of agreement between single and multiple M-line approaches for diameter, IMT, and distension, respectively. It is clearly visible that, in comparison with the outermost M-lines, the innermost M-lines showed a better agreement with the multiple M-line approach in terms of limits of agreements derived from Bland-Altman analysis. Moreover, as depicted in Figures 3D–F, central M-lines yielded better reproducibility (lower CVs) compared to the outermost M-lines.

### Effect of Averaging Multiple M-Lines

**Figure 4:**
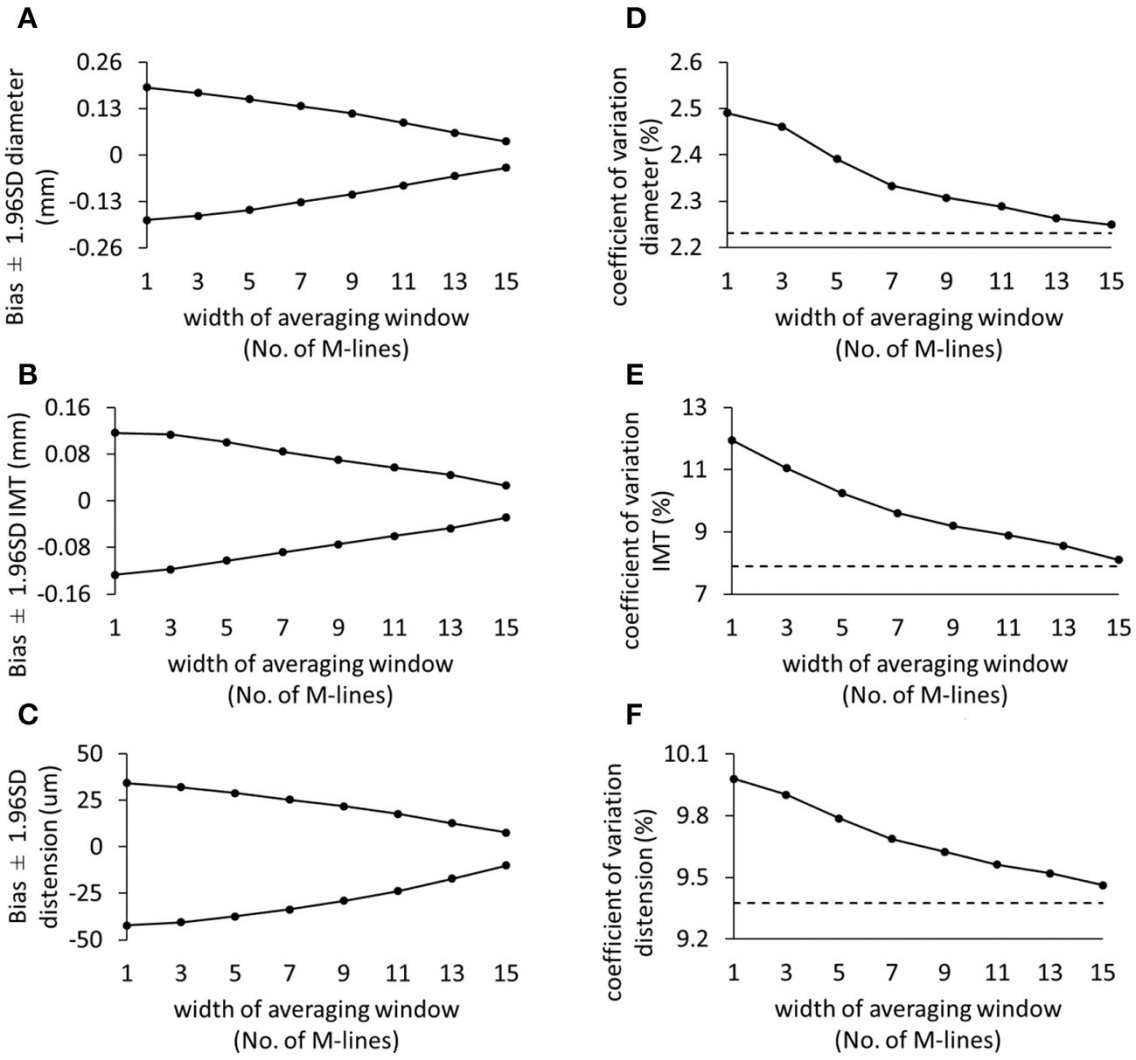
The effect of increasing the spatial averaging window on the validity **(A–C)** and reproducibility coefficient of variation **(D–F)** for diameter, intima-media thickness, and distension, respectively. The dashed lines represent the reproducibility of the multiple M-line method (i.e., including all 17 M-lines). SD, standard deviation; IMT, intima-media thickness.

The spatial averaging window starts at the middle line and extends toward the edges in steps of 2 lines. It is clearly visible from Figure 4 that including more M-lines resulted in narrower limits of agreement and lower (better) reproducibility CVs for diameter, IMT, and distension. Reproducibility curves (Figures 4D–F) have a higher slope in the first section, indicating a pronounced effect of the innermost lines on the reproducibility of carotid property measurements compared to the outermost lines.

### The Capacity of the Single M-Line Approach to Detect an Age-Related Increase in Stiffness

Distensibility coefficient and YEM values for subjects below or above 60 years of age and the inter-group differences are presented in Table 3. Both single and multiple M-line approaches showed the ability to detect an increase in stiffness with aging. Both methods revealed a significant reduction in DC as a function of age (single: difference 2.97 1/MPa, *t*-test: *p* < 0.001; multiple: difference 3.00 1/MPa, *t*-test: *p* < 0.001). Similarly, both single and multiple M-line approaches revealed a significant increase of YEM with age (single: difference 0.758 MPa, *t*-test: *p* < 0.001; multiple M-line: difference 0.756 MPa, *t*-test: *p* < 0.001).

**Table 3:** Feasibility of detecting age-related changes in stiffness.

		**Single M-line**	**Multiple M-line**	***p*-value**
Distensibility coefficient (1/MPa)	<60 y (44%)	17.00	16.82	–
	≥60 y (56%)	14.00	13.85	–
	difference	−3.00	−2.97	0.71
Young's elastic modulus (MPa)	<60 y (44%)	0.638	0.641	–
	≥60 y (56%)	0.758	0.756	–
	difference	+0.120	+0.115	0.64

*p-value shown is for the difference between single and multiple M-line approaches*.

The capability to detect the age-associated increase in stiffness was comparable for the single and multiple M-line approaches (DC: 3.00 vs. 2.97 1/MPa, ANOVA, *p* = 0.71; YEM: 0.120 vs. 0.115 MPa, ANOVA, *p* = 0.71).

## Discussion

In the present study, we evaluated the performance of single M-line ultrasound for estimating CCA properties taking multiple M-line as reference. Our evaluation showed that, although using a single M-line tends to generate less reproducible measures, the Bland-Altman analysis revealed good agreement between the single and multiple M-line approaches, with no significant bias for diameter, IMT, and YEM. Despite statistically significant biases for distension and DC, these were notably lower than the reproducibility CVs and, hence, practically irrelevant. Our results indicate that a single M-line-based technique evaluates CCA properties with sufficient validity and reproducibility for clinical use and screening purposes.

The cost of WTS, in combination with the required extensive operator expertise, hampers the large-scale application of arterial properties for screening purposes. Although single M-line-based scanners are not a technical novelty (Palombo et al., 2012), their affordability, user-friendliness, and accessibility form a basis for the wider application of arterial property screening in resource-limited settings (Joseph et al., 2020a). However, because single M-line-based scanners are image-free, they miss image-based features present in multiple M-line-based scanners such as plaque detection and characterization.

In principle, using a single M-line ultrasound without an image displayed on the screen implies increased operator dependency compared to that of multiple M-line image-based tools. However, to minimize operator dependency, single M-line tools visualize the RF signal together with real-time tracking of the arterial wall. Moreover, they also provide a signal quality score to ensure that the echos are coming from the carotid walls and that the probe is perpendicular to the walls (Joseph et al., 2020a). These together act as a feedback to guide the operator to orient the probe and reduce operator dependency induced by the lack of image feedback.

This is the first study to compare the performance of a single M-line against a multiple M-line approach in a single recording performed to measure carotid artery characteristics. In an earlier study, Palombo et al. (2012) compared two different image-based devices: one with a single M-line and one with multiple M-lines. The authors reported significant biases for diameter, distension, and beta stiffness index obtained utilizing the two devices. They explained the significant bias in carotid distension by the variance in the placement of the tracking points between the two systems. We did not encounter this issue in our study, since the single and multiple M-line estimates are based on a single recording and tracking was performed on all the M-lines simultaneously with the same tracking points.

Though the differences in distension and DC between single and multiple M-line were statistically significant, the values obtained by single and multiple M-line approaches were highly correlated (*r* = 0.99, *p* < 0.001). Moreover, considering a resolution of about 20 μm of the wall-tracking algorithm (Hoeks et al., 1990), the observed distension bias of 4 μm is technically and practically irrelevant. The observed statistical differences in distension and DC are clearly due to the large sample size and consequently high study power. These differences between single and multiple M-line approaches, however, are smaller than the reproducibility of both methods and, hence, also irrelevant when measuring or monitoring a single subject. The limits of agreement of the single and multiple M-line methods do not exceed 45 μm (about twice the resolution). Despite the absence of a consensus regarding the minimal clinically relevant difference of carotid distension, we regard the limits of the agreement we found as compatible with clinical use and screening purposes.

The single M-line approach yielded comparable reproducibility with the multiple M-line method. When considered in more detail, the reproducibility of diameter, IMT, and distension are in line with previously reported values (CV range: 2.5–11.9%) (Bianchini et al., 2010; Bozec et al., 2020). The YEM obtained using a single M-line showed the most significant difference in CV (poorest reproducibility) compared to that obtained using multiple M-lines. This substantial difference originates from the variability of the IMT measurements, which is commonly reduced by considering the mean over multiple locations along the artery. Therefore, it was expected that IMT and YEM determined with the single M-line approach would have larger variability compared to the other parameters (Table 2).

We chose to use the middle M-line of 17 M-line-based recordings as a proxy for a single M-line device. The choice of the middle M-line was arbitrary and, *a priori*, had no specific reason. With an image-free device, however, the position of the single M-line relative to the CCA is unknown. Therefore, we also evaluated the performance of the other M-lines. Clearly, the M-lines on the edges exhibited wider limits of agreement and higher reproducibility CVs compared to middle lines (Figure 3). The trend of a worse performance toward the edge can be attributed to the online tracking algorithm. The employed algorithm provides real-time visual feedback on the screen to assist the operator in orienting the probe and optimizing the quality of the M-lines. The tracking feedback entails a criterion forcing the operator to have at least 60% of the M-lines optimized. In this way, the operator may accept a measurement, without a necessity to optimize outermost lines. This technique is beneficial for time-efficient use, yet it does allow for increased noise on the outermost lines. Nevertheless, including more lines in the spatial averaging window resulted in enhanced reproducibility (Figure 4).

Our study has several possible limitations. To compare the capability of single and multiple M-line approaches toward the age-associated increase in stiffness, we addressed aging effects by looking into cross-sectional data rather than longitudinal data, although this kind of analysis should ideally consider differences within the same subjects over time.

The evaluation of arterial stiffness requires an accurate assessment of the arterial diameter changes with simultaneous measurement of the PP. Limitations of our study include the use of a single value of PP to evaluate the DC and YEM for both single and multiple M-line approaches, while, in practice, each method will have different values of PP. Nevertheless, this enables a direct comparison between the two methods in the present study. In addition, the caudal-cranial probe orientation was not fully consistent among recordings, affecting line identity (i.e., swap between 1 and 17, and so on). Therefore, probe orientation was verified by means of the sign of the correlation coefficient of the linear regression line between M-line position and the corresponding dicrotic notch for all M-lines (Hermeling et al., 2009) and, where necessary, images were vertically flipped to make probe orientation consistent prior to sensitivity analysis. In this study, single and multiple M-line estimates were derived from a single scanner and, hence, were based on the same axial and temporal resolutions. In practice, however, separate devices may come with different resolutions. Since resolution largely influences the validity and reproducibility of the estimates obtained using a scanner, it should be taken into account when considering a single M-line device for screening. There exist single M-line tools with temporal and spatial resolution comparable to that of multiple M-line tools (Joseph et al., 2020a).

Another limitation is our selection of study population and setting. First, the characteristics of our population might not be representative of the targeted population. In addition, although our ultimate target is resource-constrained areas, our study context and infrastructure were those of an expert center in a western country with adequately available resources.

Some aspects regarding our study boundaries are worth discussing. First, the current study does not present an evaluation of two systems in the same population; it is instead a retrospective evaluation of two methods based on a single recording obtained using the same scanner. However, using two devices implies the presence of two procedures, so the variance induced by positioning the probe plays a significant role in the comparison. Second, the image and visual feedback are extra information that is not available in an image-free screening tool. In this regard, our proxy may be too near ideal for a single M-line device. Hence, there is a need for a next study to address the boundaries of the present study. Therefore, our next step will be to assess the performance of a single M-line image-free device against multiple M-line scanner in a screening setting.

In conclusion, single M-line ultrasound appears as a promising tool to estimate anatomical and functional common carotid artery properties with very acceptable validity and reproducibility. Based on our results, we might infer that image-free, single M-line tools could be suited for screening and population studies in low-resource settings worldwide. Whether the comparison between single and multiple M-line devices will yield similar findings requires further study.

## Data Availability Statement

The data analyzed in this study is subject to the following licenses/restrictions: the data of this study were taken from the Maastricht Study. Data are restricted by ethics and privacy of participants according to the study protocol approved by the Institutional Medical Ethical Committee and the Netherlands Health Council. The Maastricht Study Management Team (research.dms@mumc.nl) may be contacted to request data and access is granted for researchers who follow the guidelines stated in https://www.demaastrichtstudie.nl/research/data-guidelines. Requests to access these datasets should be directed to Anke Wesselius, research.dms@mumc.nl.

## Ethics Statement

The studies involving human participants were reviewed and approved by Medisch Ethische ToetsingsCommissie UM/azM. The patients/participants provided their written informed consent to participate in this study.

## Author Contributions

AM, KR, and JJ: conceptualization. AM, KR, WM, and TD: methodology. AM, KR, and BS: data analysis. AM: data processing. AM and KR: writing—original draft preparation. AM, TD, BS, RH, JJ, CS, WM, and KR: writing—review and editing. TD, WM, and KR: supervision. All authors contributed to the article and approved the submitted version.

## Funding

AM was supported by the European Union-funded Horizon 2020 project InSiDe (no. 871547). BS was supported by the European Union's Horizon 2020 research and innovation program (no. 793805).

## Conflict of Interest

The authors declare that the research was conducted in the absence of any commercial or financial relationships that could be construed as a potential conflict of interest.

## Publisher's Note

All claims expressed in this article are solely those of the authors and do not necessarily represent those of their affiliated organizations, or those of the publisher, the editors and the reviewers. Any product that may be evaluated in this article, or claim that may be made by its manufacturer, is not guaranteed or endorsed by the publisher.

## References

[B1] Alan S. Ulgen M. S. Ozturk O. Alan B. Ozdemir L. Toprak N. (2003). Relation between coronary artery disease, risk factors and intima-media thickness of carotid artery, arterial distensibility, and stiffness index. Angiology 54, 261–267. 10.1177/000331970305400301 12785018

[B2] Avolio A. Chen S.-G. Wang R.-P. Zhang C.-L. Li M.-F. O'Rourke M. (1983). Effects of aging on changing arterial compliance and left ventricular load in a northern Chinese urban community. Circulation. 68, 50–58. 10.1161/01.CIR.68.1.50 6851054

[B3] Avolio A. Deng F.-Q. Li W.-Q. Luo Y.-F. Huang Z.-D. Xing L. (1985). Effects of aging on arterial distensibility in populations with high and low prevalence of hypertension: comparison between urban and rural communities in China. Circulation 71, 202–210. 10.1161/01.CIR.71.2.202 3965165

[B4] Bianchini E. Bozec E. Gemignani V. Faita F. Giannarelli C. Ghiadoni L. (2010). Assessment of carotid stiffness and intima-media thickness from ultrasound data: comparison between two methods. J. Ultrasound Med. 29, 1169–1175. 10.7863/jum.2010.29.8.1169 20660450

[B5] Bozec E. Girerd N. Ferreira J. P. Latar I. Zannad F. Rossignol P. (2020). Reproducibility in echotracking assessment of local carotid stiffness, diameter and thickness in a population-based study (The STANISLAS Cohort Study). Artery Res. 26, 5–12. 10.2991/artres.k.200314.001

[B6] Brands P. J. Hoeks A. P. Ledoux L. A. Reneman R. S. (1997). A radio frequency domain complex cross-correlation model to estimate blood flow velocity and tissue motion by means of ultrasound. Ultrasound Med. Biol. 23, 911–20. 10.1016/S0301-5629(97)00021-5 9300995

[B7] Engelen L. Bossuyt J. Ferreira I. van Bortel L. M. Reesink K. D. Segers P.. (2015). Reference values for local arterial stiffness. Part A: carotid artery. J. Hypertens. 33, 1981–1996. 10.1097/HJH.0000000000000654 26431185

[B8] Geijselaers S. L. Sep S. J. Schram M. T. van Boxtel M. P. van Sloten T. T. Henry R. M.. (2016). Carotid stiffness is associated with impairment of cognitive performance in individuals with and without type 2 diabetes. The Maastricht Study. Atherosclerosis 253, 186–193. 10.1016/j.atherosclerosis.2016.07.912 27503567

[B9] Godia E. C. Madhok R. Pittman J. Trocio S. Ramas R. Cabral D.. (2007). Carotid artery distensibility: a reliability study. J. Ultrasound Med. 26, 1157–1165. 10.7863/jum.2007.26.9.1157 17715309 PMC2677175

[B10] Hermeling E.. (2009). Local pulse wave velocity determination: the arterial distension waveform from foot to crest (PhD thesis). Maastricht University.

[B11] Hermeling E. Reesink K. D. Kornmann L. M. Reneman R. S. Hoeks A. P. (2009). The dicrotic notch as alternative time-reference point to measure local pulse wave velocity in the carotid artery by means of ultrasonography. J. Hypertens. 27, 2028–2035. 10.1097/HJH.0b013e32832f5890 19587605

[B12] Hoeks A. P. Brands P. J. Smeets F. A. Reneman R. S. (1990). Assessment of the distensibility of superficial arteries. Ultrasound Med. Biol. 16, 121–8. 10.1016/0301-5629(90)90139-4 2183458

[B13] Hoeks A. P. Willekes C. Boutouyrie P. Brands P. J. Willigers J. M. Reneman R. S. (1997). Automated detection of local artery wall thickness based on M-line signal processing. Ultrasound Med. Biol. 23, 1017–23. 10.1016/S0301-5629(97)00119-1 9330445

[B14] Jagannathan R. Patel S. A. Ali M. K. Narayan K. V. (2019). Global updates on cardiovascular disease mortality trends and attribution of traditional risk factors. Curr. Diab. Rep. 19, 1–12. 10.1007/s11892-019-1161-2 31222515

[B15] Joseph J. Kiran R. Nabeel P. Shah M. I. Bhaskar A. Ganesh C.. (2020a). ARTSENS® Pen—portable easy-to-use device for carotid stiffness measurement: technology validation and clinical-utility assessment. Biomed. Phys. Eng. Express 6:025013. 10.1088/2057-1976/ab74ff 33438639

[B16] Joseph J. Nabeel P. Rao S. R. Venkatachalam R. Shah M. I. Kaur P. (2020b). Assessment of carotid arterial stiffness in community settings with ARTSENS®. IEEE J. Transl. Eng. Health Med. 9, 1–11. 10.1109/JTEHM.2020.3042386 33329943 PMC7732146

[B17] Karras A. Haymann J.-P. Bozec E. Metzger M. Jacquot C. Maruani G.. (2012). Large artery stiffening and remodeling are independently associated with all-cause mortality and cardiovascular events in chronic kidney disease. Hypertension 60, 1451–1457. 10.1161/HYPERTENSIONAHA.112.197210 23090769

[B18] Kawasaki T. Sasayama S. Yagi S.-I. Asakawa T. Hirai T. (1987). Non-invasive assessment of the age related changes in stiffness of major branches of the human arteries. Cardiovasc. Res. 21, 678–687. 10.1093/cvr/21.9.678 3328650

[B19] Laurent S. Boutouyrie P. Asmar R. Gautier I. Laloux B. Guize L.. (2001). Aortic stiffness is an independent predictor of all-cause and cardiovascular mortality in hypertensive patients. Hypertension 37, 1236–1241. 10.1161/01.HYP.37.5.1236 11358934

[B20] Laurent S. Cockcroft J. Van Bortel L. Boutouyrie P. Giannattasio C. Hayoz D.. (2006). Expert consensus document on arterial stiffness: methodological issues and clinical applications. Eur. Heart J. 27, 2588–2605. 10.1093/eurheartj/ehl254 17000623

[B21] Mattace-Raso F. U. van der Cammen T. J. Hofman A. van Popele N. M. Bos M. L. Schalekamp M. A.. (2006). Arterial stiffness and risk of coronary heart disease and stroke: the Rotterdam Study. Circulation 113, 657–663. 10.1161/CIRCULATIONAHA.105.555235 16461838

[B22] Meinders J. M. Brands P. J. Willigers J. M. Kornet L. Hoeks A. P. (2001). Assessment of the spatial homogeneity of artery dimension parameters with high frame rate 2-D B-mode. Ultrasound Med. Biol. 27, 785–94. 10.1016/S0301-5629(01)00351-9 11516538

[B23] Meinders J. M. Hoeks A. P. (2004). Simultaneous assessment of diameter and pressure waveforms in the carotid artery. Ultrasound Med. Biol. 30, 147–154. 10.1016/j.ultrasmedbio.2003.10.014 14998666

[B24] Meinders J. M. Kornet L. Hoeks A. P. (2003). Assessment of spatial inhomogeneities in intima media thickness along an arterial segment using its dynamic behavior. Am. J. Physiol. Heart Circ. Physiol. 285, H384–H391. 10.1152/ajpheart.00729.2002 12637358

[B25] Palombo C. Kozakova M. Guraschi N. Bini G. Cesana F. Castoldi G.. (2012). Radiofrequency-based carotid wall tracking: a comparison between two different systems. J. Hypertens. 30, 1614–1619. 10.1097/HJH.0b013e328354dd44 22688262

[B26] Rodbard D.. (1974). Statistical quality control and routine data processing for radioimmunoassays and immunoradiometric assays. Clin. Chem. 20, 1255–1270. 10.1093/clinchem/20.10.1255 4370388

[B27] Schram M. T. Sep S. J. van der Kallen C. J. Dagnelie P. C. Koster A. Schaper N.. (2014). The Maastricht Study: an extensive phenotyping study on determinants of type 2 diabetes, its complications and its comorbidities. Eur. J. Epidemiol. 29, 439–451. 10.1007/s10654-014-9889-0 24756374

[B28] Steinbuch J. Hoeks A. P. Hermeling E. Truijman M. T. Schreuder F. H. Mess W. H. (2016). Standard b-mode ultrasound measures local carotid artery characteristics as reliably as radiofrequency phase tracking in symptomatic carotid artery patients. Ultrasound Med. Biol. 42, 586–595. 10.1016/j.ultrasmedbio.2015.07.030 26525651

[B29] Wijnands J. M. Boonen A. van Sloten T. T. Schram M. T. Sep S. J. Koster A.. (2015). Association between serum uric acid, aortic, carotid and femoral stiffness among adults aged 40–75 years without and with type 2 diabetes mellitus: the Maastricht Study. J. Hypertens. 33, 1642–1650. 10.1097/HJH.0000000000000593 26136069

[B30] Willekes C. Hoeks A. P. Bots M. L. Brands P. J. Willigers J. M. Reneman R. S. (1999). Evaluation of off-line automated intima-media thickness detection of the common carotid artery based on M-line signal processing. Ultrasound Med. Biol. 25, 57–64. 10.1016/S0301-5629(98)00138-0 10048802

[B31] World Health Organization. (2021). Cardiovascular Diseases (CVDs). World Health Organization.Available online at: https://www.who.int/news-room/fact-sheets/detail/cardiovascular-diseases-(cvds) (accessed September 22, 2021).

[B32] Yuan C. Wang J. Ying M. (2016). Predictive value of carotid distensibility coefficient for cardiovascular diseases and all-cause mortality: a meta-analysis. PLoS One 11:e0152799. 10.1371/journal.pone.0152799 27045958 PMC4821582

[B33] Zhou T. L. Henry R. M. A. Stehouwer C. D. A. van Sloten T. T. Reesink K. D. Kroon A. A. (2018). Blood pressure variability, arterial stiffness, and arterial remodeling. Hypertension 72, 1002–1010. 10.1161/HYPERTENSIONAHA.118.11325 30354707

